# Central Obesity Is Associated With an Increased Rate of Multisite Pain in Older Adults

**DOI:** 10.3389/fpubh.2022.735591

**Published:** 2022-06-14

**Authors:** Cara Dimino, Sergio L. Teruya, Kevin D. Silverman, Thelma J. Mielenz

**Affiliations:** Department of Epidemiology, Columbia University Mailman School of Public Health, New York, NY, United States

**Keywords:** multisite pain, central adiposity, older adults, waist circumference, NHATS

## Abstract

**Objective:**

Central obesity has been associated with several adverse health events, but little research exists about the longitudinal effects of central obesity on multisite pain. The purpose of this study was to assess if central obesity, as measured by waist circumference measurement, was associated with an increased rate of having multisite pain among older adults aged 65 years and older.

**Design:**

The National Health and Aging Trends Study is a longitudinal cohort study initiated in 2011 and intended to be representative of Medicare beneficiaries in the contiguous United States.

**Methods:**

There were 7,145 community-dwelling participants included in this study. Data for this study were collected annually between 2011 and 2018. Researchers assessed if waist circumference risk level was associated with an increased rate ratio of multisite pain. Weighted data were used in a multivariable generalized estimating equation model that used a log link specified with a Poisson distribution.

**Results:**

Participants with high-risk waist circumferences (98 cm or greater for women and 109 cm or greater for men) had a 11% higher rate of multisite pain than those with low-risk waist circumferences [rate ratio (*RR*) 1.11, 95% *CI*: 1.07–1.15] adjusting for gender, age, race, education, probable major depression, arthritis, and multimorbidity count.

**Conclusion:**

As measured by waist circumference, central adiposity is associated with multisite pain in older adults. While more research is needed, reducing waist circumference may prove beneficial in reducing the burden of multisite pain.

## Introduction

Two of the largest burdens on older adult populations are pain and central adiposity. These factors are associated with increased health risks and healthcare costs as well as decreased quality of life (1). Pain is associated with reduced physical function, socialization, and sleep quality (2). In addition, it commonly co-occurs with fatigue (3). Centrally accumulated body fat is considered a component of metabolic syndrome and is specifically linked to insulin resistance, coronary heart disease, cerebrovascular disease, and all-cause mortality (4). Studies indicate that central obesity is linked to several pain outcomes in the older adult population (5–8).

In a study of 7,601 adults aged 65 years and older, 52.9% reported being bothered by pain in the last month and 74.9% of those adults reported multiple sites of pain (9). The prevalence of pain reporting was highest among those with obesity (9). A cross-sectional study of 407 older adults found that those with central obesity had nearly double the odds of chronic pain prevalence as well as increased pain severity and frequency compared with those without central adiposity (5). While the temporality of the association could not be determined, it has provided a foundation for additional research (5).

Central adiposity has not yet been studied as an exposure in a longitudinal study in older adults observing pain outcomes. In this study, waist circumference will serve as a proxy measure of central adiposity, as it has been established as an appropriate measure for central adipose tissue in women and men (10). Understanding how waist circumference affects pain outcomes over time can help focus public health efforts. The present study aimed to explore how central obesity was associated with multisite pain experiences in older adults. We hypothesize that larger waist circumferences are associated with increased rates of multisite pain.

## Methods

### Data Source

The National Health and Aging Trends Study (NHATS), funded by the National Institute of Aging in the United States, is a prospective cohort study that enrolled 8,245 adults aged 65 years and older to observe late-life functioning trends (11). This longitudinal cohort is intended to represent Medicare beneficiaries in the contiguous United States (11). Researchers conducted home interviews and physical examinations with participants annually since the Round 1 baseline in 2011 (11).

### Study Population

**Figure 1:**
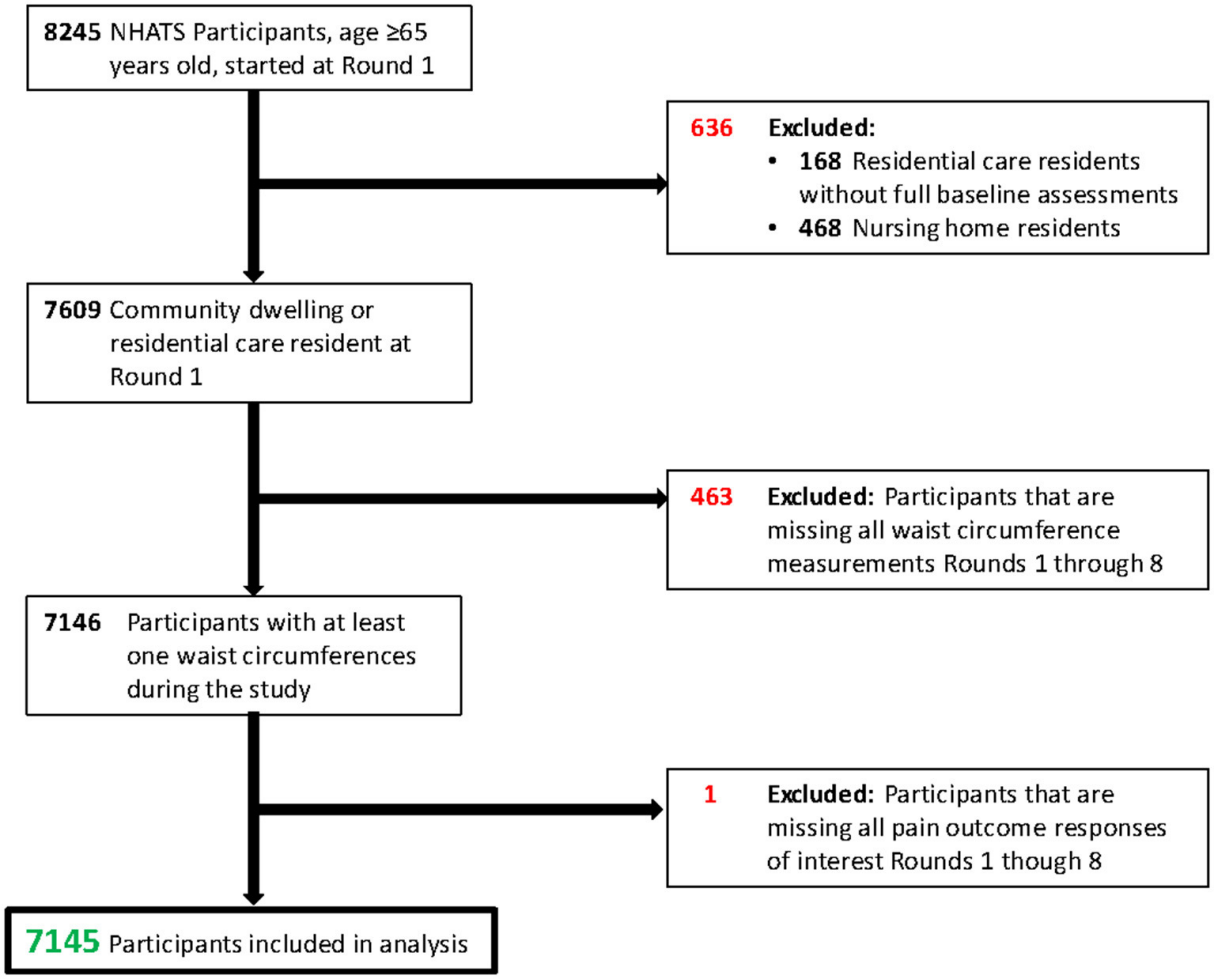
Population flow diagram.

This study uses data from the Round 1 baseline through Round 8, which were collected in 2018. Participants included community-dwelling and residential care residents that completed the baseline assessments and questionnaires. Participants were required to have at least one waist circumference measurement from Round 1 to Round 8 to be included in the study. The analysis also required that a pain outcomes assessment was done for at least one round of the study, and pain outcome measures were done for respondents indicating that they were being bothered by pain (as shown in Measures section below). After applying all exclusion criteria, the final study population was 7,145 (Figure 1). Informed consent was received in the primary data collection done by Johns Hopkins. This secondary data analysis was approved by the Columbia Irving Medical Center Institutional Review Board (AAA57372).

### Measures

#### Waist Circumference

Waist circumference will serve as a proxy measure of central adiposity, as it has been established as a convenient proxy measure for visceral adipose tissue in women and men (10). Waist circumference was measured by NHATS research staff using a flexible measuring tape at Rounds 1 through 8 (12). Depending on the capabilities of the participant, measurements were taken while the participant was standing up, sitting, or lying down (12). Measurements were taken at the naval level after a normal breath exhale (12). According to previous research, when waist circumference is adjusted for BMI, it is typically indicative of central body fat (13). Waist circumference will be explored by clinically significant cut-offs for low- and high-risk circumferences for men and women as a marker for central fat after adjusting for BMI.

The current World Health Organization (WHO) criteria for central adiposity, as measured by waist circumference, are 102 cm or greater in men and 88 cm or greater in women. These criteria have been validated in all adult populations, but due to the changes in anatomy during aging, it was important to develop more relevant clinical cut points for waist circumference risk in an older adult population (8). A previous study did this in a population of adults age 70 years and older and evaluated the most effective waist circumference cutoff values for assessing risk based on health-related outcomes (8). Prospective data on 2,232 older adult participants identified that the cutoffs most likely predicted outcomes, such as pain, mobility limitations, knee osteoarthritis, cardiovascular disease, and diabetes, were approximately 109 cm for men and 98 cm for women, so those values will be utilized here (8).

#### Self-Report of Multisite Pain

Information about participants' pain experiences was collected in the Sensory Impairments and Symptoms questionnaire (14). Researchers asked participants, “In the last month, have you ever been bothered by pain?” If participants responded no, they were not asked more specific questions about the pain. If participants responded yes, they were also asked if they had pain in their back, hip, knee, foot, hand, wrist, shoulder, head, neck, arm, leg, stomach, or other specific areas in the last month (14). Multisite pain refers to pain in more than one location, as previously described in another NHATS study (9).

#### Covariates

Characteristics previously found to be associated with pain in older adults were included as covariates in this study (9). Analyses will examine the effects of gender, defined as male or female. Models will control for age groups, which were grouped as ages 65–69, 70–74, 75–79, 80–84, 85–89, and 90 years or older at the time of the survey. The race is grouped by non-Hispanic white, non-Hispanic black, Hispanic, and others. Education was categorized as <9 years, 9–11 years, high school graduate, some college/vocational, college graduate, or masters or professional degree. Depressive symptoms were captured by the Patient Health Questionnaire-2, which asked “Over the last month, how often have you (a) had little interest or pleasure in doing things, and (b) felt down, depressed, or hopeless?” Responses were scored as 0 = not at all; 1 = several days; 2 = more than half the days; and 3 = nearly every day. A sum of both of the questions being of >3 was used to indicate probable major depression (15). Smokers are classified as never or ever smokers, with an ever smoker referring to someone that ever smoked regularly, being at least a cigarette a day for at least a month, during their lifetime. Obesity is defined as those with a body mass index (BMI) equal to or greater than 30. BMI was calculated from self-reported height and weight values. Comorbidities, such as arthritis, osteoporosis, a broken or fractured hip after age 50 years, and dementia or Alzheimer's disease, are included in the analyses. A multimorbidity count was created by adding the total number of chronic conditions reported by the participant, such as previous heart attack, diabetes, lung disease, history of a stroke, and non-skin cancer (6, 16). Multimorbidity was categorized as having either zero, one, or two or more chronic conditions, not including conditions examined separately. All covariates are based on self-reported responses during an in-person interview with a researcher, in which the researchers asked participants if a doctor ever notified them of the listed diagnoses.

### Statistical Analysis

Baseline multisite pain status and covariates were examined by waist circumference risk level using chi-squared tests. Covariates were assessed for collinearity with other covariates as well as for linearity for the outcome or association with an increased rate of multisite pain to determine how to incorporate them in the model, if appropriate at all.

A generalized estimating equation (GEE) model accounting for the NHATS sampling weights assessed if time-varying waist circumference measured by a low-risk level vs. a high-risk level is associated with an increased rate of having multisite pain. The log link function was specified with a Poisson distribution. This model was employed to account for the data clustering caused by repeated measures from the same participant over time (16). It also accounted for participants missing one or more time points of pain status responses. The adjusted models included time-varying covariates, such as age, probable major depression, and a multimorbidity count. All other included variables, such as gender, race/ethnicity, and education, were considered time-invariant. When building the model, covariates were removed one at a time, and the coefficient for waist circumference risk was monitored for a change of 10%, which would indicate confounding (17). Gender was assessed for modifying the relationship by testing the interaction term between gender and circumference in the model.

All analyses were performed using SAS Studio version 3.8 (SAS Institute, Cary, NC, USA) except for the models, which were run in Stata Version 16.0 (Stata Corp, College Station, TX, USA). For the final models in Stata, we accounted for clustering in both the unadjusted and adjusted models and specified the following: a log link, an exchangeable working correlation matrix, and robust SEs (16, 18, 19). We used the Round 8 replicate weights created by NHATS so that the proportions and measures of association were representatives of Medicare beneficiaries; the sample sizes are unweighted (20).

## Results

Table 1 describes baseline demographics, physical and mental health measures, and pain characteristics of the NHATS cohort used in this study. Study sample weights were accounted for in the weighted analyses. The study population was predominantly female (56.2%) and non-Hispanic whites (69.3%). Bothersome pain in the last month was reported by 52.7% of individuals, and 47.8% of participants met the definition of multisite pain. Of those that did not have multisite pain, 30.0% of participants did not have any pain sites and 22.2% had only one pain site. This cohort at baseline was 27.3% obese and 41.6% of participants had waist circumferences that were considered high risk based on their gender. Arthritis was the most common comorbidity (55.4%).

**Table 1:** 2011 baseline characteristics of national health and aging trends study participants, for study of central adiposity and pain.

**Characteristic**	***n***	**%**	**Weighted *%***
Total	7,145		
**Gender**			
Male	3,014	42.2	43.8
Female	4,131	57.8	56.2
**Bothered by pain**			
No	3,315	46.4	47.3
Yes	3,828	53.6	52.7
**Pain sites**			
0	2,144	30.0	30.2
1	1,585	22.2	22.6
2+ (Multisite)	3,416	47.8	47.2
**Waist circumference risk**			
Low	3,925	58.4	58.7
High	2,792	41.6	41.3
**Obese**			
No – BMI <30	5,039	72.7	71.8
Yes – BMI ≥30	1,895	27.3	28.2
**Age group**			
65–69	1,372	19.2	28.6
70–74	1,528	21.4	25.4
75–79	1,437	20.1	19.1
80–84	1,406	19.7	14.5
85–89	857	12.0	8.6
90+	545	7.6	3.8
**Race/Ethnicity**			
NH, White	4,917	69.3	81.6
NH, Black	1,540	21.7	8.0
Hispanic	429	6.1	6.8
Other	205	2.9	3.5
**Education**			
<9 years	893	12.5	10.0
<9–12 years	995	14.0	11.4
High school graduate	1,947	27.5	27.4
Some college/vocational	1,735	24.5	26.5
College graduate	828	11.7	13.2
Advanced degree	692	9.8	11.5
**Probable major depression**			
No	6,625	93.4	93.8
Yes	467	6.6	6.2
**Smoking**			
Never Smoker	3,508	49.1	52.8
Ever Smoker	3,633	50.9	47.2
Arthritis	3,950	55.4	53.4
Osteoporosis	1,437	20.2	21.0
Fractured hip after age 50	326	4.6	3.7
Dementia or Alzheimer's	308	4.3	3.2
**Comorbidities**			
Heart attack	1,065	14.9	13.7
Diabetes	1,807	25.3	23.8
Lung disease	1,093	15.3	15.5
Stroke	794	11.1	9.5
Cancer	1,826	25.6	25.8

Table 2 describes the bivariate association of waist circumference risk with all covariates assessed in this study. The low waist circumference risk is <109 cm in men or <98 cm in women. At baseline, those with high-risk waist circumferences had significantly more multisite pain than those with low-risk waist circumferences (*p* < .0001). Those with a high-risk waist circumference were also significantly more likely to be bothered by pain, women, obese people, and non-Hispanic whites. Further, having an education of high school or less, probable major depression, and a 2+ multimorbidity count were all associated with high-risk waist circumference. Smoking history, osteoporosis, dementia, and having a fractured hip after age of 50 years were not significantly associated with a higher waist circumference risk level.

**Table 2:** 2011 baseline characteristics of national health and aging trends study participants by waist circumference (WC) risk level, for study of central adiposity and pain.

**Variables, % (*n*)**	**Low risk WC**	**High risk WC**	***p^a^***
Total	58.72 (3,925)	41.28 (2,792)	
**Multisite pain**			
No	59.6	43.2	< .0001
Yes	40.4	56.8	
**Gender**			
Male	49.1	37.2	< .0001
Female	50.9	62.8	
**Bothered by pain**			
No	52.9	40.1	< .0001
Yes	47.1	59.9	
**Obesity**			
No – BMI <30	94.4	40.0	< .0001
Yes – BMI ≥30	5.6	60.0	
**Age group**			
65–74	52.6	56.9	< .0001
75–84	34.4	32.6	
85+	13.0	10.5	
**Race/Ethnicity**			
NH, White	82.9	80.7	< .0001
NH, Black	6.7	9.5	
Hispanic	6.1	7.5	
Other	4.3	2.3	
**Education**			
High school or less	45.7	52.5	< .0001
Some college	26.0	27.3	
Bachelor's +	28.3	20.2	
**Smoking**			
No	46.6	48.1	.2806
Yes	53.4	51.9	
**Depressive symptoms**			
No	94.7	93.1	.0154
Yes	5.3	6.9	
**Arthritis**			
No	52.7	38.0	< .0001
Yes	47.3	62.0	
**Osteoporosis**			
No	79.0	79.4	.7210
Yes	21.0	29.6	
**Fractured Hip after age 50**			
No	96.4	96.3	.8609
Yes	3.6	3.7	
**Dementia or Alzheimer's**			
No	96.9	97.3	.3420
Yes	3.1	2.7	
**Multimorbidity count**			
0	46.5	33.3	< .0001
1	35.7	38.9	
2+	17.8	27.8	

Missing values for pain responses were examined over time and by the WC risk group to inform any potential bias. At any given round, no more than 10.1% of the sample was missing a pain response for a group. The breakdown of missing pain response data by the WC risk group at each round is presented in Table 3.

**Table 3:** 2011 missing pain responses of national health and aging trends study participants by waist circumference (WC) risk level, for study of central adiposity and pain.

**Round, % missing**	**Low risk WC**	**High risk WC**
1	0.0%	0.0%
2	4.9%	4.5%
3	8.1%	7.5%
4	9.3%	7.4%
5	9.4%	6.1%
6	10.1%	7.5%
7	9.8%	9.7%
8	9.8%	9.1%

The final model adjusted for cluster sampling and the following covariates: gender, age, race, education, probable major depression, arthritis, and multimorbidity count. In this model, having a high-risk waist circumference increased the rate of having multisite pain by 10.6% [rate ratio (*RR*) 1.106, 95% *CI*: 1.066–1.147], adjusting for all covariates (Table 4). The interaction term between waist circumference risk level and gender was not significant in the model (*p* = .786) and therefore, gender did not modify the association between waist circumference and having multisite pain on the multiplicative scale. Having arthritis was a confounder of the relationship between waist circumference risk level and having multisite pain.

**Table 4:** Waist circumference high and low risk levels and the rate of having multisite pain among national health and aging trends study participants 2011–2018.

	**Multisite pain**	
	**Unadjusted *n* = 2,722 (Observations in GEE = 20,202)**	**Adjusted^a^ *n* = 2,707 (Observations in GEE = 19,968)**
	**Rate ratio (95% CI)**	**Rate ratio (95% CI)**
**Waist circumference**		
Low risk level	Reference^b^	Reference^b^
High risk level	1.147 (1.105–1.190)	1.106 (1.066–1.147)

a *Multivariable Poisson regression generalized estimating equation model adjusted for gender, age, race, education, probable major depression and mulitmorbidity count (0, 1 or 2 or more with 0 as the referent), accounting for clustering of repeated measures from participants over time*.

b *Reference for High risk waist circumference group*.

Body mass index was not surprisingly found to be collinear with waist circumference, and thus we did not include BMI or obesity, determined by BMI, in the model. However, we did test BMI as a dichotomous variable (BMI <30 or ≥30) in the model and found that while it did confound the relationship between waist circumference risk and having multisite pain, waist circumference was still significant at *p* < .0001 when in the fully adjusted model (data not reported).

## Discussion

The findings from this cohort study indicate that there is a significant relationship between waist circumference risk level and the rate of having multisite pain. We found that those with a high-risk waist circumference had a 11% increased rate of having multisite pain compared to those with low-risk waist circumferences, even after adjusting for gender, age, race, education, probable major depression, arthritis, and multimorbidity count. Notably, women in this study were more likely than men to have multisite pain in the adjusted model. These results are consistent with findings from previous cohort and cross-sectional studies that have assessed the relationship between central adiposity and pain in the older adult population (5–8, 21).

While BMI was not included in the final model due to collinearity with waist circumference, it is interesting that when tested in the fully adjusted model, waist circumference was still significant when BMI was in the model. A prior study assessed waist circumference and BMI together in a model assessing pain outcomes but found that the association of BMI and pain outcomes was stronger than that of waist circumference and BMI (7). However this study does not address the claim of other researchers that including waist circumference and BMI in a model together could give a better description of fat distribution, namely, central adiposity (13).

Further research needs to be done on the relationship between waist circumference and BMI in the older adult population to determine the most appropriate way to model central adiposity. Our study is strengthened by employing 8 years of data from the NHATS longitudinal cohort. The waist circumference measure was administered using a rigorous and validated research protocol, which instills confidence in the measure. The pain was ascertained by a researcher during an in-person interview that presented very clear and concrete questions about pain location. If there was any recall bias about pain, it would probably be a non-differential overestimation of multisite pain. Since researchers asked participants about pain in several listed locations, a participant might have been more likely to say “yes” to a location of pain than if they were asked to list where pain bothers them. This study has several limitations. NHATS does not have a comprehensive pain assessment which makes it difficult to determine the extent to which an individual is in pain. This study is also limited in that many variables are self-reported, such as height, weight, and comorbidities. For instance, BMI should be viewed with caution because height and weight were self-reported and may be underestimated. Ideally, future studies would consider additional confounders, such as daily physical activity and also pain medications used by participants (13). Furthermore, while only a small percentage of participants were missing pain responses at each round, findings may be biased as a result. In most rounds, the proportion of missing responses for each group is relatively similar. However, there are several rounds where the low-risk WC group is missing a slightly greater percentage of pain responses, and the computed rate ratios may be biased.

The use of a multimorbidity count is also a limitation of this study, as it possibly oversimplifies the impacts of certain comorbidities on multisite pain. Patients with different comorbidities but with an equal number of them are categorized in the same multimorbidity count group. Except for depressive symptoms, arthritis, osteoporosis, and dementia or Alzheimer's disease, comorbidities (e.g., heart attack, diabetes, lung disease, stroke, and cancer) are examined as an aggregate count. Therefore, we cannot comment on the relationship between specific comorbidities and multisite pain. Because of the limited sample size, we chose to employ this simplified method. However, we did choose to separately examine comorbidities that have been examined by or found to be associated with pain in previous studies (9, 22, 23). For example, Vennu et al. found that obesity was associated with multisite pain in the lower extremities in a population with arthritis (22). In terms of waist circumference, Costa et al. (23) reported an adjusted odds ratio (*OR*) of 2.03 (1.57–2.63) for waist circumference and multisite chronic pain while adjusting for depression .

One of the challenges of pain research is that pain can be measured in a number of different ways (e.g., by intensity, severity, duration, and location) (24). The pain PROMIS measures, created by the National Institute of Health to target specific pain-related domains, are changing this but currently, there is not one common scale that is used across most epidemiological research. Heim et al. (7) performed a prospective study on a cohort of 2,000 older adults enrolled in the Longitudinal Aging Study Amsterdam. This study assessed pain using a modified Nottingham Health Profile that seeks to understand overall body pain, and they found two- to three-fold increased odds of incident pain after 6 years for those in the highest waist circumference quartile vs. the lowest (7). This association is much stronger than the one found here, which can be attributed to study differences, but also to the measurement of a different aspect of pain.

## Conclusions

Measuring methods aside, it will become increasingly important to mitigate pain by all means possible for populations as they age. This study supports that central adiposity, as measured by waist circumference, is associated with an increased rate of multisite pain in older adults. While more research is needed, reducing waist circumference may prove beneficial in reducing the burden of multisite pain. Obesity remains a debated topic in older adults because the changes to the body during aging have made it harder to identify if obesity is even clinically relevant in this population (8, 21). It seems that central adiposity and loss of fat-free mass are more clinically relevant for older adults (21). While weight circumference is not a perfect measure of central adiposity, it might be more clinically relevant to assess an older adults' waist circumference risk vs. their scale weight. Moreover, finding more evidence to support age-appropriate waist circumference cut points in older adults will help researchers establish clinically significant levels that will make screening and identifying people at risk easier.

## Data Availability Statement

Publicly available datasets were analyzed in this study. This data can be found at: https://nhats.org.

## Ethics Statement

The studies involving human participants were reviewed and approved by the Columbia Irving Medical Center Institutional Review Board (AAA57372). Written informed consent for participation was not required for this study in accordance with the national legislation and the institutional requirements.

## Author Contributions

TM conceptualized the study and provided supervision of the study. CD and TM conducted the data analysis, the methodology, the data visualization, and wrote the original draft. ST and KS provided an in-depth review of the manuscript and assistance in the scientific writing, as well as contribution to data interpretation. All authors contributed to the article and approved the submitted version.

## Funding

The National Center for Injury Prevention and Control, Centers for Disease Control and Prevention to Columbia University (R49 CE002096-01). Its contents are solely the responsibility of the authors and do not necessarily represent the official views of the centers for Disease Control and Prevention. U.S. Department of Health and Human Services, National Institutes of Health, National Institute on Aging (NIA U01AG032947).

## Conflict of Interest

The authors declare that the research was conducted in the absence of any commercial or financial relationships that could be construed as a potential conflict of interest.

## Publisher's Note

All claims expressed in this article are solely those of the authors and do not necessarily represent those of their affiliated organizations, or those of the publisher, the editors and the reviewers. Any product that may be evaluated in this article, or claim that may be made by its manufacturer, is not guaranteed or endorsed by the publisher.
